# Cardiorenal Protection in Type 2 Diabetes: A Systematic Review Comparing Glucagon-Like Peptide-1 Receptor Agonists and Sodium-Glucose Cotransporter-2 Inhibitors

**DOI:** 10.7759/cureus.116039

**Published:** 2026-09-10

**Authors:** Philip Branigan, Vo Nhu Y Duong, Stephanie Branigan

**Affiliations:** 1 Cardiology, State University of New York (SUNY) Downstate College of Medicine, Brooklyn, USA; 2 Cardiovascular Research, Methodist Hospitals, Merrillville, USA; 3 Internal Medicine, Peconic Bay Medical Center, Riverhead, USA

**Keywords:** cardiovascular disease, chronic kidney disease, glp-1 receptor agonists, sglt2 inhibitors, type 2 diabetes mellitus

## Abstract

Type 2 diabetes mellitus (T2DM) is associated with substantial cardiovascular and renal morbidity and premature mortality. Sodium-glucose cotransporter-2 (SGLT2) inhibitors and glucagon-like peptide-1 (GLP-1) receptor agonists reduce cardiorenal events beyond their glucose-lowering effects. This systematic review compared cardiovascular, heart failure, kidney, mortality, metabolic, and safety outcomes of the two classes of medications and evaluated evidence for complementary use. Randomized controlled trials, systematic reviews and meta-analyses, and large comparative observational studies of adults with type 2 diabetes reporting relevant cardiovascular, renal, mortality, metabolic, or safety outcomes were eligible. Glycemia-only studies without relevant clinical outcomes, pediatric and preclinical studies, case reports, editorials, and noninformative reports were excluded. PubMed/MEDLINE, Embase, and Cochrane CENTRAL were searched from January 2010 through August 30, 2026, supplemented by Google Scholar and backward and forward citation searching. Risk of bias was assessed using RoB 2 for randomized trials, ROBINS-I for nonrandomized comparative studies, and AMSTAR 2 for systematic reviews and meta-analyses. Certainty of evidence was assessed at the outcome level using the Grading of Recommendations Assessment, Development and Evaluation (GRADE) framework. Because of heterogeneity across populations, interventions, outcomes, and study designs, results were evaluated using a structured narrative synthesis rather than a de novo meta-analysis.

Thirty-eight studies were included, comprising 14 randomized controlled trials, 12 nonrandomized comparative studies, and 12 systematic reviews or meta-analyses. SGLT2 inhibitors showed particularly consistent benefit for heart failure and kidney outcomes, whereas GLP-1 receptor agonists showed strong atherosclerotic cardiovascular benefit and greater weight reduction. Certainty was moderate for the principal comparative cardiovascular and kidney outcomes and lower for real-world comparative effectiveness and combination therapy. Evidence supported potentially complementary cardiorenal effects with combined use, although superiority of combination therapy over appropriately selected monotherapy was not established. Safety profiles differed, with genital infections, volume depletion, and rare diabetic ketoacidosis more closely associated with SGLT2 inhibitors and gastrointestinal adverse effects more common with GLP-1 receptor agonists. Interpretation was limited by heterogeneity in study populations, interventions, outcome definitions, follow-up periods, and study designs, residual confounding in observational evidence, and the possibility of publication and selective-reporting bias. Overall, the findings support individualized treatment selection according to cardiovascular and renal risk, heart failure, weight goals, safety, tolerability, and access, with combined use considered when complementary benefits are clinically appropriate

## Introduction and background

Type 2 diabetes mellitus (T2DM) is a progressive metabolic disorder associated with substantial cardiovascular and kidney morbidity and premature mortality. T2DM accounts for approximately 90-95% of diabetes cases and affects an estimated 589-828 million adults worldwide, representing approximately 11-14% of the adult population [1-2]. Cardiovascular disease affects approximately one-third of adults with T2DM, while diabetes remains an important contributor to chronic kidney disease (CKD) and kidney failure [1-2]. Population-level modeling further demonstrates the substantial clinical and economic burden associated with myocardial infarction, stroke, heart failure, and end-stage kidney disease in people with T2DM [3]. Cardiovascular outcomes have therefore become an important component of diabetes treatment and drug evaluation, shifting attention from glycemic control alone toward whether glucose-lowering therapies can also reduce major cardiovascular events, heart failure, kidney disease progression, and mortality.

Over the past decade, sodium-glucose cotransporter-2 (SGLT2) inhibitors and glucagon-like peptide-1 (GLP-1) receptor agonists have expanded the goals of T2DM treatment beyond glycemic control. Both classes improve metabolic parameters and have demonstrated cardiovascular and kidney benefits, but their mechanisms and strongest clinical effects differ. SGLT2 inhibitors reduce renal glucose reabsorption in the proximal tubule, resulting in increased urinary glucose excretion. GLP-1 receptor agonists enhance glucose-dependent insulin secretion, suppress glucagon secretion, delay gastric emptying, and promote weight loss [4-5]. These differences provide a biological basis for their distinct and potentially complementary clinical effects.

Landmark cardiovascular outcome trials established the cardiorenal effects of both drug classes. EMPA-REG OUTCOME demonstrated reductions in cardiovascular mortality and hospitalization for heart failure with empagliflozin, while Canagliflozin Cardiovascular Assessment Study (CANVAS) and DECLARE-TIMI 58 provided additional evidence for cardiovascular, heart failure, and kidney benefits with SGLT2 inhibition [6-8]. Subsequent trials expanded these findings beyond traditional cardiovascular outcome populations. DAPA-HF and EMPEROR-Reduced demonstrated benefits in patients with heart failure with reduced ejection fraction [9-10], while DELIVER extended evidence for dapagliflozin to patients with mildly reduced or preserved ejection fraction [11]. Dedicated kidney outcome trials, including CREDENCE, DAPA-CKD, and EMPA-KIDNEY, further established the role of SGLT2 inhibitors in slowing CKD progression and reducing cardiorenal events [12-14].

GLP-1 receptor agonists have demonstrated a somewhat different pattern of benefit. LEADER and SUSTAIN-6 showed reductions in major adverse cardiovascular events (MACE) with liraglutide and semaglutide, respectively [15-16]. In addition to their cardiovascular effects, GLP-1 receptor agonists provide clinically important reductions in body weight and have increasingly demonstrated kidney benefits. The FLOW trial provided dedicated kidney outcome evidence for semaglutide in patients with T2DM and CKD, further expanding the role of this class in cardiorenal risk reduction [17]. As this evidence accumulated, treatment recommendations increasingly incorporated cardiovascular, heart failure, kidney, and metabolic risk into the selection of glucose-lowering therapy rather than relying primarily on glycemic targets [18-21]. More recent randomized and observational evidence has also examined GLP-1 receptor agonists and SGLT2 inhibitors in combination, raising the possibility that their differing mechanisms and outcome profiles may provide complementary benefits [22-28].

Despite the growing evidence supporting both classes of medications, important questions remain regarding their relative and complementary roles in patients with different cardiovascular, heart failure, kidney, and metabolic risk profiles. Much of the randomized evidence was generated in placebo-controlled cardiovascular, heart failure, or kidney outcome trials rather than direct comparisons between SGLT2 inhibitors and GLP-1 receptor agonists. Comparative observational studies and meta-analyses provide additional evidence, but differences in study populations, outcome definitions, follow-up periods, and study designs complicate interpretation. Evidence regarding combination therapy is also evolving, particularly for cardiovascular and kidney outcomes beyond those established for either class alone.

This systematic review addresses these gaps by integrating randomized controlled trials, comparative observational studies, and systematic reviews and meta-analyses within a single clinically focused framework. It incorporates recent dedicated cardiovascular, heart failure, and kidney outcome evidence while also evaluating metabolic effects, safety, real-world comparative effectiveness, and the emerging evidence for complementary use of both classes. By considering study design and risk of bias when interpreting differences across the evidence base, this review aims to clarify where the benefits of SGLT2 inhibitors and GLP-1 receptor agonists overlap, where they differ, and how these differences may inform individualized treatment decisions in adults with T2DM.

## Review

Methods

Review Design and Registration

This systematic review was conducted and reported in accordance with the Preferred Reporting Items for Systematic Reviews and Meta-Analyses (PRISMA) 2020 statement (Appendix 1). The review evaluated cardiovascular, heart failure, kidney, mortality, metabolic, and safety outcomes associated with SGLT2 inhibitors and GLP-1 receptor agonists in adults with T2DM, including evidence regarding their complementary use. Because substantial clinical and methodological heterogeneity was expected across study populations, interventions, comparators, outcome definitions, follow-up periods, and study designs, a structured narrative synthesis was used rather than a de novo meta-analysis.

The review was retrospectively registered with the Open Science Framework (OSF) on August 30, 2026 (registration DOI: 10.17605/OSF.IO/PRNYA). Earlier narrative-review work preceded registration. The subsequent systematic-review conversion included an updated literature search, deduplication, independent screening, documentation of full-text exclusion reasons, structured data extraction, risk-of-bias assessment, and PRISMA 2020 reporting.

Eligibility Criteria

Eligibility criteria were defined according to the population, intervention, comparator, outcomes, and study design framework. Studies involving adults with T2DM were eligible if they evaluated an SGLT2 inhibitor, a GLP-1 receptor agonist, or the use of both classes and reported relevant cardiovascular, heart failure, kidney, mortality, metabolic, or safety outcomes. Eligible comparators included placebo or usual care, active comparators, the alternative drug class, and concomitant use of the other class.

Eligible study designs included randomized controlled trials, systematic reviews and meta-analyses, and large comparative observational studies. Studies were excluded if they focused solely on glycemic outcomes without reporting relevant clinical outcomes, involved pediatric populations or preclinical models, were case reports or editorials, represented duplicate or overlapping reports without unique data, or did not provide adequate information for extraction. When multiple reports described the same underlying study population, reports were linked to avoid double-counting.

Clinical guidelines, consensus statements, narrative reviews, cost-effectiveness analyses, and descriptive or survey studies cited elsewhere in the manuscript for background, clinical context, implementation, cost, or access were not considered part of the systematic evidence synthesis.

No statistical sample-size calculation was performed because all studies meeting the prespecified eligibility criteria were considered for inclusion. The final number of included studies was therefore determined by the systematic search and screening process rather than by a predetermined sample size.

Information Sources and Search Period

Systematic searches were conducted in PubMed/MEDLINE, Embase, and Cochrane CENTRAL. Google Scholar was used as an additional source, and backward and forward citation searching was performed to identify additional potentially eligible studies. Searches were run on August 30, 2026, and covered studies published from January 1, 2010, through August 30, 2026.

January 1, 2010, was selected as the starting date to capture the contemporary evidence base leading to and following the major cardiovascular and cardiorenal outcome programs for SGLT2 inhibitors and GLP-1 receptor agonists. The search period was intended to encompass the development of clinically relevant cardiovascular, heart failure, kidney, metabolic, and safety evidence for these classes rather than the broader historical literature on glucose-lowering therapies.

Search Strategy

Three complementary searches were conducted within each primary bibliographic database: an SGLT2 inhibitor class-specific search, a GLP-1 receptor agonist class-specific search, and a direct comparison or combination search. Separate class-specific searches were used so that potentially eligible studies were not required to mention both drug classes.

Search terminology incorporated population terms, alternative class terminology, individual drug names, and relevant clinical outcomes. Population terms included "type 2 diabetes," "type 2 diabetes mellitus," and "T2DM." SGLT2 inhibitor terms included "SGLT2," "SGLT-2," "SGLT2 inhibitor," "SGLT2 inhibitors," "sodium-glucose cotransporter 2 inhibitor," "sodium-glucose cotransporter 2 inhibitors," empagliflozin, canagliflozin, dapagliflozin, and ertugliflozin. GLP-1 receptor agonist terms included "GLP-1," "GLP1," "GLP-1 receptor agonist," "GLP-1 receptor agonists," "glucagon-like peptide-1 receptor agonist," "glucagon-like peptide-1 receptor agonists," liraglutide, semaglutide, dulaglutide, exenatide, lixisenatide, and albiglutide. Outcome terms included cardiovascular disease, major adverse cardiovascular events (MACE), atherosclerotic cardiovascular disease (ASCVD), heart failure, heart failure hospitalization, stroke, mortality, cardiovascular death, renal and kidney outcomes, CKD, estimated glomerular filtration rate (eGFR), kidney failure, kidney disease progression, safety, and adverse events. Direct comparison and combination searches additionally included HbA1c and weight.

In PubMed/MEDLINE, search terms were restricted to Title/Abstract fields. Corresponding searches in Embase and Cochrane CENTRAL were adapted to their respective title and abstract syntax. The complete database-specific search strategies, including the exact Boolean search strings, search terms, field restrictions, search dates, date limits, and retrieval counts for each search, are provided in Appendix 2.

The database searches identified 15,774 records in PubMed/MEDLINE, 27,908 in Embase, and 2,587 in Cochrane CENTRAL, yielding 46,269 database records before deduplication. Google Scholar was searched separately for SGLT2 inhibitors, GLP-1 receptor agonists, and direct comparison or combination evidence. Because of the large number of Google Scholar results, the first 200 results from each of the three searches were screened, for a total of 600 Google Scholar records. Backward and forward citation searching identified an additional 47 records.

Study Selection

Records identified through the searches were combined and deduplicated before screening. Two reviewers independently screened titles and abstracts according to the prespecified eligibility criteria. Records with uncertain eligibility were retained for full-text assessment rather than excluded during initial screening.

Potentially eligible reports then underwent independent full-text assessment by two reviewers. One primary reason for exclusion was recorded for each report excluded at the full-text stage. Disagreements were resolved through discussion and re-review of the source report. Decisions that remained unresolved were adjudicated by the third author. Multiple reports arising from the same underlying study were identified and linked to avoid double-counting.

**Figure 1:**
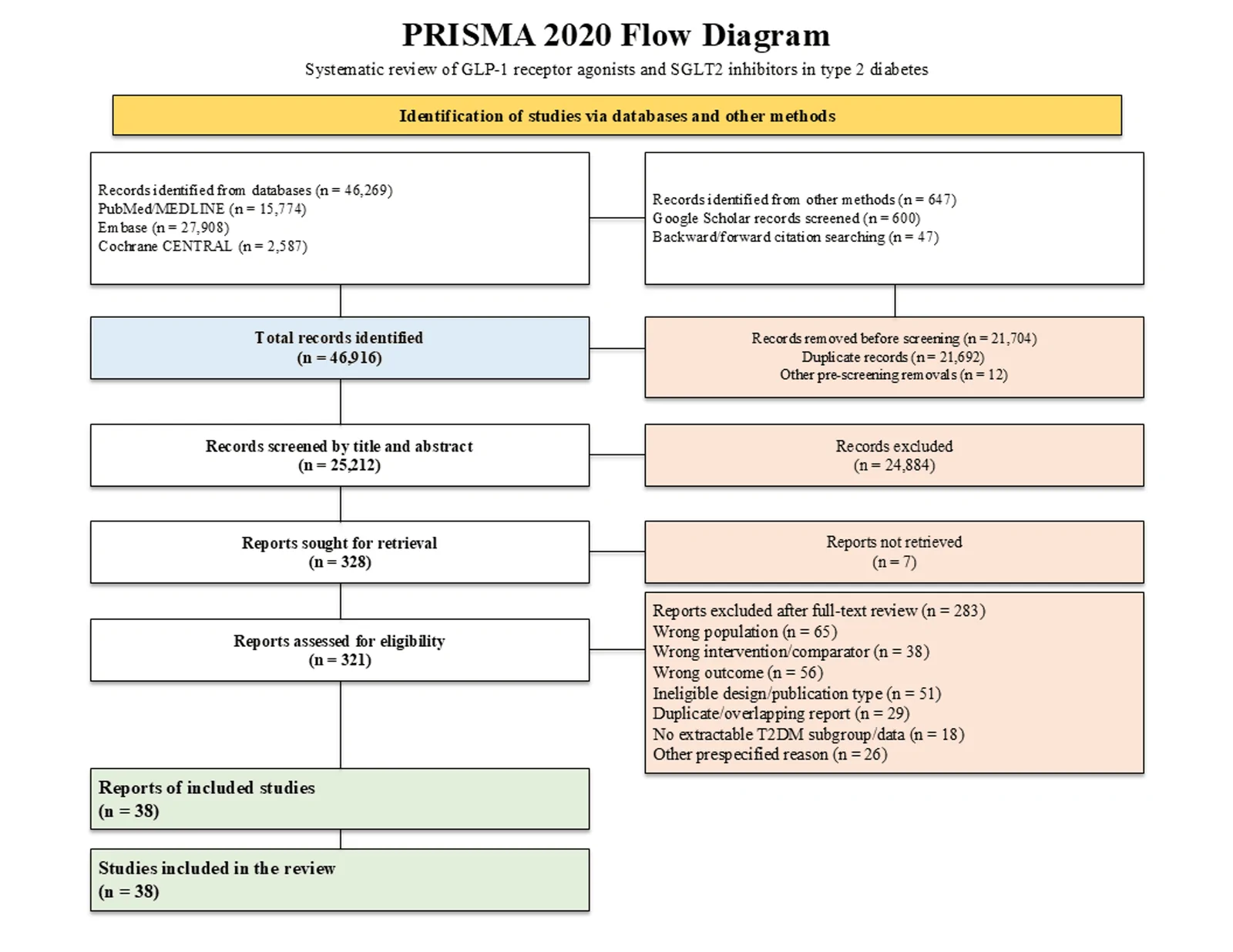
PRISMA 2020 flow diagram of study identification, screening, eligibility, and inclusion. PRISMA 2020 flow diagram depicting the identification, screening, full-text assessment, and inclusion of studies in the systematic review evaluating GLP-1 RAs and SGLT2 inhibitors in T2DM. Records were identified through PubMed/MEDLINE, Embase, Cochrane CENTRAL, Google Scholar, and backward and forward citation searching. After duplicate and other pre-screening removals, 25,212 records underwent title and abstract screening, 321 reports were assessed for eligibility, and 38 studies were included in the final review. Reasons for full-text exclusion are shown in the diagram. The figure was created using Microsoft PowerPoint (Microsoft Corporation, Redmond, WA). GLP-1 RAs: Glucagon-like peptide-1 receptor agonists; PRISMA: Preferred Reporting Items for Systematic Reviews and Meta-Analyses; SGLT2: Sodium-glucose cotransporter 2; T2DM: Type 2 diabetes mellitus.

The study-selection process was documented using a PRISMA 2020 flow diagram. The searches identified 46,916 records from databases and other methods. After the removal of 21,692 duplicate records and 12 other records before screening, 25,212 records underwent title and abstract screening. Of these, 24,884 were excluded, and 328 reports were sought for retrieval. Seven reports could not be retrieved, leaving 321 reports for full-text eligibility assessment. Following the exclusion of 283 reports, 38 studies met the eligibility criteria and were included in the systematic review. Reasons for exclusion at the full-text stage are presented in the PRISMA 2020 flow diagram (Figure 1).

Data Extraction

Data were extracted using a structured approach. Extracted variables included study citation, study design, setting, sample size, participant characteristics, intervention and comparator, dose when relevant, duration of follow-up, baseline ASCVD, heart failure and CKD status, reported outcomes, effect estimates and 95% confidence intervals when available, adverse events, funding information, and information required for risk-of-bias assessment.

Prespecified randomized trial estimates and adjusted estimates from observational studies were prioritized. Reported effect measures, time points, denominators, and confidence intervals were retained, and unreported values were not inferred or imputed. One reviewer performed structured data extraction, and a second reviewer independently verified key outcomes and risk-of-bias judgments. Disagreements were resolved through re-review of the source study and consensus, with third-author adjudication when necessary.

Risk-of-Bias Assessment

Risk of bias was assessed according to study design. Randomized controlled trials were evaluated using the Cochrane Risk of Bias 2 (RoB 2) tool. Nonrandomized comparative studies were evaluated using the Risk of Bias In Non-randomized Studies of Interventions (ROBINS-I) tool. Systematic reviews and meta-analyses included as evidence sources were evaluated using AMSTAR 2. Individual study-level assessments are provided in Appendix 3.

Risk-of-bias assessments were incorporated into the interpretation of the evidence rather than treating all studies as equally informative. Greater weight was given to randomized evidence and higher-quality systematic reviews when findings differed across study designs. Observational evidence was interpreted primarily as complementary evidence regarding comparative effectiveness, safety, and real-world generalizability. A sensitivity analysis was performed by excluding nonrandomized studies judged to be at serious risk of bias and systematic reviews or meta-analyses rated as critically low confidence to determine whether their exclusion materially changed the principal conclusions. Results of the risk-of-bias assessments and sensitivity analysis are reported in the Results section.

Data Synthesis

Because of clinical and methodological heterogeneity across study populations, interventions, comparators, outcome definitions, follow-up periods, and study designs, a de novo meta-analysis was not performed. Findings were instead evaluated using a structured narrative synthesis organized according to prespecified clinical outcome domains, including MACE and ASCVD, heart failure, kidney outcomes, cardiovascular and all-cause mortality, stroke, metabolic effects, safety, and combination therapy.

Randomized controlled trials and higher-quality systematic reviews and meta-analyses were given greater weight when evaluating efficacy. Comparative observational studies were used primarily to assess comparative effectiveness, safety, and generalizability in routine clinical practice. Within each outcome domain, the direction and magnitude of reported effects, precision, consistency across studies, directness of the evidence, heterogeneity, and risk of bias were considered. Statistical significance alone was not used to determine the strength of a conclusion, and causal conclusions were not drawn solely from observational evidence.

When findings differed across study designs, conclusions were based primarily on evidence with stronger methodological design and lower risk of bias. Discordant observational findings were interpreted in the context of potential residual confounding and differences in study populations rather than pooled with clinically or methodologically dissimilar evidence. Results were presented narratively by prespecified clinical outcome domain and summarized in Table 1 [29-42], with individual study-level risk-of-bias assessments presented in Appendix 3 and outcome-level certainty assessments presented in Appendix 4. No formal subgroup analysis or meta-regression was performed.

**Table 1:** Comparative clinical outcomes of SGLT2 inhibitors and GLP-1 receptor agonists in type 2 diabetes. Summary of the comparative glycemic, weight, cardiovascular, kidney, safety, and combination-therapy findings across studies included in this systematic review. The “Predominant evidence type” column indicates the principal study design supporting each summarized outcome. Statements reflect the overall direction and consistency of the included evidence and should not be interpreted as direct head-to-head estimates unless supported by comparative studies. Reference numbers correspond to the manuscript reference list. GLP-1: Glucagon-like peptide-1; HbA1c: Glycated hemoglobin; MACE: Major adverse cardiovascular events; RCT: Randomized controlled trial; SGLT2: Sodium-glucose cotransporter 2.

Outcome	SGLT2 inhibitors	GLP-1 receptor agonists	Predominant evidence type	References
Glycemic control	Clinically meaningful HbA1c reduction	Clinically meaningful HbA1c reduction, generally greater with potent long-acting agents	Systematic reviews/meta-analyses of RCTs	[29-30]
Weight	Modest weight reduction	Greater weight reduction overall	Systematic reviews/meta-analyses of RCTs	[29-30]
MACE and atherosclerotic cardiovascular outcomes	Cardiovascular benefit demonstrated; MACE benefit generally less prominent than heart failure benefit	Consistent reduction in MACE in cardiovascular outcome trials, with particular benefit for atherosclerotic outcomes	RCTs; systematic review/meta-analysis of RCTs	[6-8,15,16,31]
Heart failure hospitalization	Consistent reduction across cardiovascular and dedicated heart failure trials	No comparably consistent reduction demonstrated	RCTs; systematic review/meta-analysis	[9-11,31]
Stroke	No consistent class-specific reduction demonstrated	Greater benefit for stroke, particularly nonfatal stroke, in comparative evidence	RCTs; systematic review/meta-analysis	[15,16,31]
Kidney disease progression	Strong and consistent benefit across dedicated kidney outcome trials	Kidney benefit supported by cardiovascular trials and strengthened by dedicated FLOW evidence	RCTs; systematic review/meta-analysis	[12-14,17,38]
Cardiovascular mortality	Reduction demonstrated in selected high-risk populations	Reduction demonstrated in selected populations; no consistent between-class superiority established	RCTs; systematic review/meta-analysis	[6,15,29]
All-cause mortality	Benefit demonstrated in selected populations	Benefit demonstrated in selected populations; comparative superiority remains uncertain	Systematic review/network meta-analysis of RCTs	[29]
Key adverse effects	Genital infections, volume depletion, and rare diabetic ketoacidosis	Gastrointestinal adverse effects are more common	RCT-based systematic reviews/meta-analyses; comparative observational evidence	[29-30,34,42]
Combination therapy	Effects appear preserved with concomitant GLP-1 receptor agonist use	Effects appear preserved with concomitant SGLT2 inhibitor use; evidence suggests potentially complementary effects, but superiority over appropriately selected monotherapy is not established	RCT subgroup/meta-analytic evidence; comparative observational studies	[22-28]

Publication and Selective-Reporting Bias

The possibility of publication and selective-reporting bias was considered when interpreting the evidence. Because a de novo meta-analysis of sufficiently homogeneous studies was not performed, formal statistical assessment using funnel plots or regression-based tests was not considered appropriate. Publication and selective-reporting bias were therefore evaluated qualitatively in relation to the completeness of the evidence base, consistency of findings across evidence sources, and availability of evidence from different study designs. The use of multiple bibliographic databases, Google Scholar, and backward and forward citation searching was intended to improve the completeness of study identification.

Certainty of Evidence

Certainty of evidence was assessed at the outcome level using the Grading of Recommendations Assessment, Development and Evaluation (GRADE) framework. The domains considered were risk of bias, inconsistency, indirectness, imprecision, and publication bias. Randomized and nonrandomized evidence streams were considered according to their contribution to each outcome. Nonrandomized comparative studies were appraised using ROBINS-I and entered the GRADE assessment at high certainty, with subsequent downgrading when residual confounding or other limitations warranted. Certainty was categorized as high, moderate, low, or very low.

Because most randomized trials compared an active therapy with placebo or usual care rather than directly comparing SGLT2 inhibitors with GLP-1 receptor agonists, indirectness was specifically considered when evaluating between-class conclusions. For example, certainty regarding the kidney benefit of each class compared with placebo was distinguished from certainty regarding the relative kidney effects of the two classes. Publication bias was considered as a potential downgrading domain when the evidence depended on a small number of direct studies, including the combination-therapy evidence. The outcome-level GRADE assessments and reasons for downgrading are presented in Appendix 4.

Results

Study Selection

The searches identified 46,916 records. After removal of 21,704 records before screening, 25,212 records underwent title and abstract screening, of which 24,884 were excluded. Of 328 reports sought for retrieval, seven could not be retrieved, leaving 321 reports for full-text assessment. Following full-text review, 283 reports were excluded, and 38 studies met the eligibility criteria for inclusion in the systematic review. Reasons for full-text exclusion and the complete study-selection process are presented in the PRISMA 2020 flow diagram (Figure 1).

Characteristics of Included Studies

The 38 studies included in the systematic evidence synthesis comprised 14 randomized controlled trials, 12 nonrandomized comparative studies, and 12 systematic reviews or meta-analyses. The evidence base included cardiovascular outcome trials, dedicated heart failure and kidney outcome trials, comparative observational studies, and studies evaluating concomitant use of SGLT2 inhibitors and GLP-1 receptor agonists.

Risk of Bias

Risk of bias varied across the included evidence and by study design. Among 14 randomized controlled trials assessed using RoB 2, 11 were judged to be at low risk of bias, three had some concerns, and none were judged to be at high risk of bias. Among 12 nonrandomized comparative studies assessed using ROBINS-I, eight were judged to be at moderate risk of bias and four at serious risk of bias; none were judged to be at low or critical risk. Residual confounding was an important consideration when interpreting observational comparisons. Among 12 systematic reviews and meta-analyses evaluated using AMSTAR 2, four were rated as high confidence, four as moderate confidence, two as low confidence, and two as critically low confidence. Individual study-level assessments are provided in Appendix 2.

A sensitivity analysis excluding the four nonrandomized studies at serious risk of bias and the two systematic reviews rated as critically low confidence did not materially change the principal conclusions, as the direction and approximate magnitude of the cardiovascular and kidney benefits were preserved.

Certainty of Evidence

Certainty of evidence varied by outcome. Certainty was rated as moderate for comparative effects on MACE, heart failure hospitalization, nonfatal stroke, kidney disease progression or kidney failure, and all-cause and cardiovascular mortality. Indirectness was the principal limitation for most comparative efficacy outcomes because direct randomized comparisons between SGLT2 inhibitors and GLP-1 receptor agonists were limited. For kidney outcomes, the moderate rating applied specifically to the between-class comparison; certainty that each class reduces kidney disease progression compared with placebo was higher.

Certainty was low for comparative effectiveness in real-world subgroups because of residual confounding and serious risk of bias in part of the nonrandomized evidence. Certainty was also low for severe gastrointestinal adverse effects because of risk of bias and imprecision. Evidence that SGLT2 inhibitors increase genital infections was rated as high certainty. Cardiovascular and kidney outcomes with combination therapy were rated as low certainty because of indirectness, the contribution of observational evidence, and suspected publication bias related to the small number of direct combination studies. Detailed outcome-level GRADE assessments and reasons for downgrading are presented in Appendix 3.

Cardiovascular Outcomes

Randomized cardiovascular outcome trials demonstrated cardiovascular benefit with both SGLT2 inhibitors and GLP-1 receptor agonists, although the pattern of benefit differed between the two classes. EMPA-REG OUTCOME demonstrated reductions in cardiovascular mortality and hospitalization for heart failure with empagliflozin, while CANVAS and DECLARE-TIMI 58 provided additional randomized evidence supporting cardiovascular benefit with SGLT2 inhibition [6-8]. GLP-1 receptor agonist trials, including LEADER and SUSTAIN-6, demonstrated reductions in MACE, with evidence supporting benefit for atherosclerotic cardiovascular outcomes and stroke [15-16].

Systematic reviews and meta-analyses comparing the two classes further supported differences in their cardiovascular effects. Both classes reduced composite MACE compared with placebo, while direct comparative evidence did not demonstrate a significant between-class difference in composite MACE. Component-level effects differed, with GLP-1 receptor agonists showing greater benefit for atherosclerotic outcomes, including stroke, and SGLT2 inhibitors showing more consistent benefit for heart failure outcomes [29-31]. Because much of the randomized evidence was derived from separate placebo-controlled trials rather than direct head-to-head randomized comparisons, certainty in the between-class MACE comparison was rated as moderate because of indirectness. Comparative observational studies provided additional direct evidence but remained subject to residual confounding [32-37].

Heart Failure Outcomes

Heart failure benefit was most consistently demonstrated with SGLT2 inhibitors. Cardiovascular outcome trials showed reductions in hospitalization for heart failure, and dedicated heart failure trials expanded these findings to patients with established heart failure [6-10]. DAPA-HF and EMPEROR-Reduced demonstrated benefit in patients with heart failure with reduced ejection fraction [9-10], while DELIVER demonstrated benefit with dapagliflozin in patients with mildly reduced or preserved ejection fraction [11].

GLP-1 receptor agonists demonstrated cardiovascular benefit but did not show the same consistency of benefit for heart failure outcomes. Comparative and meta-analytic evidence similarly favored SGLT2 inhibitors for the prevention of heart failure hospitalization [29,31]. Certainty in the comparative heart failure conclusion was rated as moderate because, despite strong and consistent randomized evidence supporting SGLT2 inhibitor benefit, most between-class comparisons were indirect.

Kidney Outcomes

SGLT2 inhibitors demonstrated consistent benefit across dedicated kidney outcome trials. CREDENCE showed kidney protection with canagliflozin in patients with T2DM and nephropathy, while DAPA-CKD and EMPA-KIDNEY expanded the evidence for SGLT2 inhibition across broader CKD populations [12-14]. Collectively, these randomized trials demonstrated reductions in kidney disease progression and cardiorenal events.

GLP-1 receptor agonists also demonstrated kidney benefit. Earlier cardiovascular outcome trials reported favorable kidney outcomes, and FLOW provided dedicated randomized kidney outcome evidence for semaglutide in patients with T2DM and CKD [17]. A subsequent meta-analysis of randomized trials further supported kidney and cardiovascular benefits associated with GLP-1 receptor agonists [38].

Comparative observational studies and target-trial emulations provided additional evidence regarding the relative kidney effectiveness of SGLT2 inhibitors and GLP-1 receptor agonists [27, 32-33,35-36,39]. These studies generally supported a stronger effect of SGLT2 inhibition on kidney disease progression, although the observational comparisons remained subject to residual confounding. Certainty in the between-class kidney comparison was rated as moderate because direct randomized class-to-class comparisons remain limited. This rating does not indicate uncertainty that the individual classes provide kidney benefit compared with placebo, which is supported by dedicated randomized trials.

Mortality Outcomes

Mortality findings varied according to the study population, intervention, and duration of follow-up. EMPA-REG OUTCOME demonstrated a reduction in cardiovascular mortality with empagliflozin in patients with T2DM and established cardiovascular disease [6]. Other randomized trials and meta-analyses reported cardiovascular and all-cause mortality outcomes across broader cardiovascular and kidney risk populations [29,31, 40-41].

The available evidence did not demonstrate consistent superiority of one class over the other for mortality across all populations with T2DM. Direct randomized comparisons between SGLT2 inhibitors and GLP-1 receptor agonists for mortality were limited, and comparative conclusions were therefore based largely on separate randomized trial programs, meta-analytic evidence, and observational comparisons. Certainty in the between-class mortality conclusion was rated as moderate because of this indirectness.

Metabolic Effects

Both SGLT2 inhibitors and GLP-1 receptor agonists improved glycemic outcomes. Comparative evidence generally demonstrated greater weight reduction with GLP-1 receptor agonists, while both classes produced modest reductions in blood pressure [29-30]. The magnitude of metabolic effects varied according to the individual agent, study population, treatment duration, and background therapy.

Safety and Tolerability

The two classes demonstrated distinct adverse-effect profiles. SGLT2 inhibitors were associated with increased genital infections, volume depletion, and rare diabetic ketoacidosis, whereas gastrointestinal adverse effects were more common with GLP-1 receptor agonists [29-30, 34, 42]. Rates of individual adverse events varied across studies and patient populations.

Certainty was high for the increased risk of genital infections associated with SGLT2 inhibitors, reflecting a large, consistent, and precise class-specific safety signal. Certainty regarding severe gastrointestinal adverse effects with GLP-1 receptor agonists was rated as low because of heterogeneity in event definitions and ascertainment, low event frequency, and imprecision in comparative estimates. Observational evidence provided additional information regarding safety and treatment patterns in routine clinical practice, but these findings remained subject to potential confounding and differences in baseline patient characteristics.

Combination Therapy

Evidence evaluating concomitant SGLT2 inhibitor and GLP-1 receptor agonist therapy suggested potential complementary metabolic and cardiorenal effects. Randomized and meta-analytic evidence evaluated SGLT2 inhibitor effects with and without background GLP-1 receptor agonist therapy and GLP-1 receptor agonists used as add-on therapy to SGLT2 inhibitors [22-23,25-26]. Observational studies also evaluated cardiovascular and kidney outcomes associated with combined treatment in routine clinical practice [24,27-28].

Although the overall direction of the evidence supported potential complementary benefit, superiority of combination therapy over appropriately selected monotherapy was not established. The combination-therapy literature was heterogeneous with respect to study design, treatment sequence, patient population, follow-up, and outcome definitions, and much of the direct evidence was observational. Certainty for cardiovascular and kidney outcomes with combination therapy was therefore rated as low because of indirectness, risk of bias, and suspected publication bias associated with the relatively small number of direct combination studies.

Discussion

The findings of this systematic review indicate that SGLT2 inhibitors and GLP-1 receptor agonists both provide cardiovascular and kidney benefits in adults with T2DM, but the evidence does not support treating the two classes as interchangeable. Their relative strengths differ across cardiovascular, heart failure, kidney, metabolic, and safety outcomes. These differences are summarized in Table 1 and should be interpreted in the context of study design, risk of bias, and certainty of evidence rather than by comparing effect estimates from clinically heterogeneous studies as though they were derived from direct head-to-head trials.

Cardiovascular Outcomes and Mortality

Both drug classes reduce cardiovascular risk, although the pattern of benefit differs. Randomized cardiovascular outcome trials established cardiovascular benefit with SGLT2 inhibitors, with EMPA-REG OUTCOME demonstrating reductions in cardiovascular mortality and hospitalization for heart failure with empagliflozin [6]. CANVAS and DECLARE-TIMI 58 subsequently reinforced the cardiovascular safety and cardiorenal benefits of SGLT2 inhibition [7-8]. In contrast, GLP-1 receptor agonist trials demonstrated particularly consistent effects on atherosclerotic cardiovascular outcomes. LEADER demonstrated cardiovascular benefit with liraglutide [15], while SUSTAIN-6 demonstrated a reduction in MACE with semaglutide [16]. Comparative evidence suggests component-level differences, with GLP-1 receptor agonists showing greater benefit for atherosclerotic outcomes, including stroke, and SGLT2 inhibitors showing more consistent benefit for heart failure outcomes [31].

These differences should not be interpreted as demonstrating overall cardiovascular superiority of one class. Both classes reduce composite MACE compared with placebo, while direct comparative evidence has not demonstrated a significant between-class difference in composite MACE. Certainty in the comparative MACE evidence was rated as moderate, primarily because most randomized evidence was derived from separate placebo-controlled trial programs rather than direct head-to-head comparisons.

Mortality findings are similarly less suitable for a simple between-class comparison. Mortality benefits have been demonstrated in selected high-risk populations, including the cardiovascular mortality reduction observed with empagliflozin in EMPA-REG OUTCOME [6]. However, differences in baseline cardiovascular disease, kidney disease, follow-up, individual agents, and outcome definitions limit comparison of mortality estimates across separate trial programs. The available evidence therefore does not establish consistent mortality superiority of one class over the other. Certainty in this between-class conclusion was rated as moderate because of indirectness.

Heart Failure

Heart failure is one of the clearest areas of differentiation between the two classes. The reduction in heart failure hospitalization observed in SGLT2 inhibitor cardiovascular outcome trials was reinforced by dedicated heart failure studies. DAPA-HF demonstrated benefit with dapagliflozin in patients with heart failure with reduced ejection fraction [9], and EMPEROR-Reduced provided complementary randomized evidence for empagliflozin [10]. DELIVER subsequently extended the evidence for dapagliflozin to patients with heart failure with mildly reduced or preserved ejection fraction [11].

GLP-1 receptor agonists have not demonstrated the same consistency of benefit for heart failure hospitalization. Their cardiovascular benefits appear more strongly related to atherosclerotic outcomes than to heart failure events [15, 16, 31]. The strength and consistency of the randomized heart failure evidence therefore support SGLT2 inhibition when heart failure is a predominant clinical concern. Nevertheless, certainty in the direct between-class conclusion was rated as moderate because most comparisons between SGLT2 inhibitors and GLP-1 receptor agonists remain indirect.

Kidney Outcomes

The kidney evidence has expanded considerably for both drug classes. SGLT2 inhibitors have a particularly extensive body of dedicated randomized kidney outcome evidence. CREDENCE demonstrated kidney protection with canagliflozin in patients with T2DM and nephropathy [12]. DAPA-CKD subsequently demonstrated kidney benefit with dapagliflozin across a broader CKD population [13], and EMPA-KIDNEY further expanded the evidence supporting SGLT2 inhibition in patients at risk for progressive CKD [14]. These trials provide strong evidence that SGLT2 inhibitors reduce kidney disease progression.

The kidney evidence for GLP-1 receptor agonists has also evolved beyond reductions in albuminuria. FLOW provided dedicated randomized evidence supporting kidney benefit with semaglutide in patients with T2DM and CKD [17], and subsequent meta-analytic evidence further supported kidney and cardiovascular benefits across randomized GLP-1 receptor agonist trials [38]. Accordingly, GLP-1 receptor agonists should no longer be characterized as providing only modest albuminuria-based kidney benefit.

The available evidence nevertheless differs in breadth and composition between the classes. SGLT2 inhibitors are supported by several dedicated CKD outcome programs, whereas dedicated GLP-1 receptor agonist kidney outcome evidence is more recent. Direct comparative observational studies provide additional information regarding relative kidney effectiveness [27, 32-33, 35-36, 39], but these findings require more cautious interpretation because of residual confounding.

An important distinction is the difference between certainty in the benefit of each class and certainty in the comparison between them. Dedicated randomized trials provide high certainty that each class can reduce kidney disease progression compared with placebo, but certainty in the between-class comparison was rated as moderate because direct randomized comparisons remain limited. The larger effect observed with SGLT2 inhibitors for kidney disease progression was considered a class-related difference rather than unexplained inconsistency.

Metabolic Effects and Cardiovascular Risk

Metabolic effects provide another clinically relevant distinction between the classes. Both improve glycemic control, while GLP-1 receptor agonists generally produce greater weight reduction [29-30]. Both classes can also produce modest reductions in blood pressure. These effects should be interpreted as components of broader cardiovascular risk reduction rather than substitutes for established management of hypertension, dyslipidemia, obesity, and other cardiovascular risk factors.

The metabolic profile of GLP-1 receptor agonists may be particularly relevant when obesity and atherosclerotic cardiovascular risk are prominent treatment priorities. Conversely, the heart failure and kidney effects of SGLT2 inhibitors may carry greater weight when those conditions predominate. This pattern supports individualized treatment selection based on the patient's major sources of cardiorenal and metabolic risk rather than selection based solely on glycemic efficacy.

Safety and Tolerability

The two classes have distinct adverse-effect profiles that may influence treatment selection. SGLT2 inhibitors are associated with genital infections, volume depletion, and rare diabetic ketoacidosis, whereas gastrointestinal adverse effects are more common with GLP-1 receptor agonists [29, 30]. Comparative studies in older adults also highlight differences in genitourinary events, acute kidney injury, and other adverse outcomes [34, 42].

The certainty of these safety findings was not uniform. Evidence for increased genital infections with SGLT2 inhibitors was rated as high certainty because of the large, consistent, and precise class-specific signal. In contrast, evidence regarding severe gastrointestinal adverse effects with GLP-1 receptor agonists was rated as low certainty because of heterogeneity in event definitions and ascertainment and imprecision in comparative estimates. This distinction illustrates why the presence of an adverse-effect signal and the certainty surrounding its magnitude should be considered separately.

Combination Therapy

The differing mechanisms and clinical effects of SGLT2 inhibitors and GLP-1 receptor agonists provide a rationale for combined therapy, but the strength of evidence for combination treatment is less established than the evidence supporting either class individually. The SMART-C collaborative meta-analysis found that the efficacy and safety effects of SGLT2 inhibitors were generally consistent with and without concomitant GLP-1 receptor agonist therapy [22]. Meta-analytic evidence has also evaluated GLP-1 receptor agonists added to SGLT2 inhibitor therapy [23, 25]. In the FLOW trial population, semaglutide effects were additionally examined according to concomitant SGLT2 inhibitor use [26].

Observational evidence has examined combined treatment in routine clinical practice, including cardiovascular and serious kidney outcomes [24, 27-28]. These studies provide clinically relevant evidence but are more susceptible to treatment-selection bias and residual confounding than randomized comparisons. Differences in treatment sequence, duration of combined exposure, patient characteristics, and outcome definitions further limit direct comparison across studies.

The available evidence therefore supports the possibility of complementary benefit but does not establish that combined treatment is uniformly superior to appropriately selected monotherapy. GRADE certainty for cardiovascular and kidney outcomes with combination therapy was low because of indirectness, the contribution of observational evidence, and suspected publication bias related to the relatively small number of direct combination studies. These findings support cautious consideration of combination therapy rather than assuming an additive clinical benefit for every patient.

Interpretation According to Level and Certainty of Evidence

The distinction between randomized, observational, and meta-analytic evidence is central to the interpretation of the findings. Randomized controlled trials provide the strongest evidence for causal treatment effects but generally evaluated each class against placebo or usual care rather than directly randomizing patients between SGLT2 inhibitors and GLP-1 receptor agonists. Consequently, conclusions about differences between the classes are often less direct than conclusions about the efficacy of either class individually.

Systematic reviews and meta-analyses increase precision and facilitate comparison across trial programs but remain dependent on the quality and comparability of the underlying studies. Direct comparative observational studies address an important evidence gap by evaluating the two classes within the same clinical populations, but residual confounding limits causal interpretation. A recent example is the 2026 comparative cardiovascular-effectiveness analysis by Bu et al. [37], which contributes direct real-world comparative evidence but remains observational in design.

The formal risk-of-bias assessment reflected these differences. Eleven of 14 randomized controlled trials were judged to be at low risk of bias, three had some concerns, and none were at high risk. Among the 12 nonrandomized comparative studies, eight were at moderate risk and four at serious risk. Among 12 systematic reviews and meta-analyses, four were rated as high confidence, four as moderate confidence, two as low confidence, and two as critically low confidence. Sensitivity analysis excluding the four nonrandomized studies at serious risk of bias and the two systematic reviews rated as critically low confidence did not materially alter the principal cardiovascular and kidney conclusions.

The GRADE assessment further demonstrated that certainty differed across clinical outcomes. Comparative evidence for MACE, heart failure hospitalization, nonfatal stroke, kidney outcomes, and mortality was generally rated as moderate. Indirectness was the predominant limitation for most comparative efficacy conclusions because direct randomized comparisons between the two classes were limited. Real-world comparative effectiveness was rated as low certainty because of residual confounding, including serious risk-of-bias judgments in four nonrandomized studies. These distinctions reinforce the importance of considering both the direction of an observed effect and the certainty of the evidence supporting it.

Heterogeneity and Limitations

Several sources of heterogeneity should be considered when interpreting this review. Study populations differed in baseline ASCVD, heart failure, CKD, kidney function, age, glycemic control, background therapy, and duration of follow-up. Cardiovascular and kidney composite outcomes were also defined differently across studies. In addition, the evidence included placebo-controlled randomized trials, observational head-to-head comparisons, conventional meta-analyses, and network meta-analyses. Quantitative estimates from these different designs should not be interpreted as though they arose from a single homogeneous evidence base.

Direct randomized comparisons between SGLT2 inhibitors and GLP-1 receptor agonists remain limited. This indirectness was the principal reason several comparative outcomes were rated as moderate rather than high certainty under GRADE. Some comparative conclusions therefore depend on indirect comparisons across randomized trial programs supplemented by direct observational evidence. Residual confounding remains an important limitation of the latter, particularly because prescribing decisions may be influenced by baseline cardiovascular disease, kidney function, obesity, treatment access, and other patient characteristics [43].

Publication and selective-reporting bias also cannot be excluded. Because heterogeneity did not support a de novo meta-analysis, formal funnel-plot or regression-based assessment of publication bias was not performed. Publication bias was instead considered qualitatively. Searching multiple bibliographic databases, Google Scholar, and backward and forward citation searching increased the breadth of study identification but cannot ensure that all unpublished or selectively reported evidence was identified. Suspected publication bias contributed specifically to the low-certainty GRADE assessment for combination therapy, for which the number of direct studies was relatively small.

The review was retrospectively registered during conversion of an earlier narrative review into a systematic review. Although the subsequent systematic search, independent screening, eligibility assessment, risk-of-bias evaluation, structured synthesis, and certainty assessment were documented, retrospective registration limits the ability to demonstrate that all methodological decisions preceded examination of the literature.

Clinical Implications and Implementation Challenges

Taken together, the evidence supports a risk-based approach to the selection of these medications. SGLT2 inhibitors have particularly strong evidence when heart failure or progressive CKD is a major clinical concern. GLP-1 receptor agonists provide strong atherosclerotic cardiovascular benefit and greater weight reduction, with FLOW and other recent evidence also establishing clinically important kidney protection [44-45]. The moderate certainty of many between-class comparisons should not be interpreted as uncertainty about whether the individual classes are effective, but rather as reflecting the limited availability of direct randomized comparisons between them.

For patients with residual risk across multiple domains, combined therapy may provide complementary benefit, although the evidence supporting combination therapy is less mature and was rated as low certainty. Treatment decisions should therefore consider the patient's predominant cardiovascular, heart failure, kidney, and metabolic risks together with safety, tolerability, and treatment preferences. Obstructive sleep apnea is an additional clinically relevant comorbidity in type 2 diabetes because of its association with cardiovascular and microvascular disease [46], and recent observational evidence has examined the use of incretin receptor agonists and CPAP among adults with diabetes, obesity, and obstructive sleep apnea [47].

Clinical effectiveness also depends on whether patients can initiate and continue treatment. Cost, insurance coverage, adherence, adverse effects, contraindications, route of administration, and clinician prescribing practices can influence the use of these therapies [18, 21, 48-52]. These factors may contribute to differences between efficacy observed in randomized trials and effectiveness in routine clinical practice.

## Conclusions

This systematic review provides an updated, clinically focused synthesis of the cardiovascular, heart failure, kidney, metabolic, and safety evidence for SGLT2 inhibitors and GLP-1 receptor agonists in adults with T2DM. The findings support distinct but complementary clinical strengths rather than uniform superiority of either class. SGLT2 inhibitors demonstrated particularly consistent benefits for heart failure and kidney outcomes, while GLP-1 receptor agonists demonstrated strong atherosclerotic cardiovascular benefit and greater weight reduction, with recent evidence also strengthening their role in kidney protection. Treatment selection should therefore reflect the patient's predominant cardiovascular, heart failure, kidney, and metabolic risks, as well as safety and tolerability.

This review addresses an important gap by integrating randomized controlled trials, comparative observational studies, and systematic reviews and meta-analyses within a single framework while accounting for differences in study design, risk of bias, and certainty of evidence. Although the benefits of each class are supported by substantial randomized evidence, certainty in many between-class comparisons was moderate because direct head-to-head randomized evidence remains limited. Evidence for complementary combination therapy was of lower certainty and did not establish superiority over appropriately selected monotherapy. By incorporating recent dedicated cardiorenal evidence and distinguishing established class-specific benefits from less certain comparative findings, this review provides a contemporary framework for understanding where the benefits of SGLT2 inhibitors and GLP-1 receptor agonists differ, where they overlap, and when their complementary use may be clinically appropriate.

## Appendices

Appendix 1 

**Table 2:** Completed PRISMA 2020 checklist. Completed Preferred Reporting Items for Systematic Reviews and Meta-Analyses (PRISMA) 2020 checklist for this systematic review. The checklist identifies the locations within the manuscript, figures, and supplementary materials where each PRISMA 2020 reporting item is addressed. Items not applicable to this review, including those pertaining to de novo meta-analysis or other statistical synthesis, are identified accordingly. PRISMA: Preferred Reporting Items for Systematic Reviews and Meta-Analyses; RCT: Randomized controlled trial; OSF: Open Science Framework; DOI: Digital Object Identifier.

Section and Topic	Item #	Checklist item	Location where item is reported
TITLE			
Title	1	Identify the report as a systematic review.	Title
ABSTRACT			
Abstract	2	See the PRISMA 2020 for Abstracts checklist.	Abstract
INTRODUCTION			
Rationale	3	Describe the rationale for the review in the context of existing knowledge.	Introduction and Background
Objectives	4	Provide an explicit statement of the objective(s) or question(s) the review addresses.	Introduction and Background, final paragraph
METHODS			
Eligibility criteria	5	Specify the inclusion and exclusion criteria for the review and how studies were grouped for the syntheses.	Methods: Eligibility Criteria; Data Synthesis
Information sources	6	Specify all databases, registers, websites, organisations, reference lists and other sources searched or consulted to identify studies. Specify the date when each source was last searched or consulted.	Methods: Information Sources and Search Period; Search Strategy
Search strategy	7	Present the full search strategies for all databases, registers and websites, including any filters and limits used.	Methods: Search Strategy; Supplementary Table 1
Selection process	8	Specify the methods used to decide whether a study met the inclusion criteria of the review, including how many reviewers screened each record and each report retrieved, whether they worked independently, and if applicable, details of automation tools used in the process.	Methods: Study Selection
Data collection process	9	Specify the methods used to collect data from reports, including how many reviewers collected data from each report, whether they worked independently, any processes for obtaining or confirming data from study investigators, and if applicable, details of automation tools used in the process.	Methods: Data Extraction
Data items	10a	List and define all outcomes for which data were sought. Specify whether all results that were compatible with each outcome domain in each study were sought (e.g. for all measures, time points, analyses), and if not, the methods used to decide which results to collect.	Methods: Eligibility Criteria; Data Extraction; Data Synthesis
	10b	List and define all other variables for which data were sought (e.g. participant and intervention characteristics, funding sources). Describe any assumptions made about any missing or unclear information.	Methods: Data Extraction
Study risk of bias assessment	11	Specify the methods used to assess risk of bias in the included studies, including details of the tool(s) used, how many reviewers assessed each study and whether they worked independently, and if applicable, details of automation tools used in the process.	Methods: Risk-of-Bias Assessment; Supplementary Table 2
Effect measures	12	Specify for each outcome the effect measure(s) (e.g. risk ratio, mean difference) used in the synthesis or presentation of results.	Methods: Data Extraction; Data Synthesis
Synthesis methods	13a	Describe the processes used to decide which studies were eligible for each synthesis (e.g. tabulating the study intervention characteristics and comparing against the planned groups for each synthesis (item #5)).	Methods: Eligibility Criteria; Data Synthesis
	13b	Describe any methods required to prepare the data for presentation or synthesis, such as handling of missing summary statistics, or data conversions.	Methods: Data Extraction
	13c	Describe any methods used to tabulate or visually display results of individual studies and syntheses.	Methods: Data Synthesis; Table 1; Supplementary Tables 2 and 3
	13d	Describe any methods used to synthesize results and provide a rationale for the choice(s). If meta-analysis was performed, describe the model(s), method(s) to identify the presence and extent of statistical heterogeneity, and software package(s) used.	Methods: Review Design and Registration; Data Synthesis
	13e	Describe any methods used to explore possible causes of heterogeneity among study results (e.g. subgroup analysis, meta-regression).	Methods: Data Synthesis (no formal subgroup analysis or meta-regression performed)
	13f	Describe any sensitivity analyses conducted to assess robustness of the synthesized results.	Methods: Risk-of-Bias Assessment
Reporting bias assessment	14	Describe any methods used to assess risk of bias due to missing results in a synthesis (arising from reporting biases).	Methods: Publication and Selective-Reporting Bias
Certainty assessment	15	Describe any methods used to assess certainty (or confidence) in the body of evidence for an outcome.	Methods: Certainty of Evidence; Supplementary Table 3
RESULTS			
Study selection	16a	Describe the results of the search and selection process, from the number of records identified in the search to the number of studies included in the review, ideally using a flow diagram.	Results: Study Selection; Figure 1 (PRISMA 2020 flow diagram)
	16b	Cite studies that might appear to meet the inclusion criteria, but which were excluded, and explain why they were excluded.	Results: Study Selection; Figure 1 (full-text exclusion reasons)
Study characteristics	17	Cite each included study and present its characteristics.	Results: Characteristics of Included Studies; Results outcome subsections; Supplementary Table 2
Risk of bias in studies	18	Present assessments of risk of bias for each included study.	Results: Risk of Bias; Supplementary Table 2
Results of individual studies	19	For all outcomes, present, for each study: (a) summary statistics for each group (where appropriate) and (b) an effect estimate and its precision (e.g. confidence/credible interval), ideally using structured tables or plots.	Results: Cardiovascular, Heart Failure, Kidney, Mortality, Metabolic, Safety, and Combination Therapy subsections; Table 1
Results of syntheses	20a	For each synthesis, briefly summarise the characteristics and risk of bias among contributing studies.	Results: Characteristics of Included Studies; Risk of Bias; outcome-specific Results subsections
	20b	Present results of all statistical syntheses conducted. If meta-analysis was done, present for each the summary estimate and its precision (e.g. confidence/credible interval) and measures of statistical heterogeneity. If comparing groups, describe the direction of the effect.	Not applicable: no de novo meta-analysis or other statistical synthesis was performed
	20c	Present results of all investigations of possible causes of heterogeneity among study results.	Not applicable: no formal subgroup analysis or meta-regression was performed; heterogeneity was considered qualitatively
	20d	Present results of all sensitivity analyses conducted to assess the robustness of the synthesized results.	Results: Risk of Bias (sensitivity analysis)
Reporting biases	21	Present assessments of risk of bias due to missing results (arising from reporting biases) for each synthesis assessed.	Results: Certainty of Evidence; Safety and Tolerability; Combination Therapy; Discussion: Heterogeneity and Limitations
Certainty of evidence	22	Present assessments of certainty (or confidence) in the body of evidence for each outcome assessed.	Results: Certainty of Evidence; Supplementary Table 3
DISCUSSION			
Discussion	23a	Provide a general interpretation of the results in the context of other evidence.	Discussion: Cardiovascular Outcomes and Mortality through Clinical Implications and Implementation Challenges
	23b	Discuss any limitations of the evidence included in the review.	Discussion: Heterogeneity and Limitations; Interpretation According to Level and Certainty of Evidence
	23c	Discuss any limitations of the review processes used.	Discussion: Heterogeneity and Limitations
	23d	Discuss implications of the results for practice, policy, and future research.	Discussion: Clinical Implications and Implementation Challenges; Conclusions
OTHER INFORMATION			
Registration and protocol	24a	Provide registration information for the review, including register name and registration number, or state that the review was not registered.	Methods: Review Design and Registration; Abstract
	24b	Indicate where the review protocol can be accessed, or state that a protocol was not prepared.	Methods: Review Design and Registration (OSF registration DOI 10.17605/OSF.IO/PRNYA)
	24c	Describe and explain any amendments to information provided at registration or in the protocol.	Methods: Review Design and Registration (retrospective registration and systematic-review conversion described)
Support	25	Describe sources of financial or non-financial support for the review, and the role of the funders or sponsors in the review.	Abstract; Funding/Support statement
Competing interests	26	Declare any competing interests of review authors.	Competing Interests declaration
Availability of data, code and other materials	27	Report which of the following are publicly available and where they can be found: template data collection forms; data extracted from included studies; data used for all analyses; analytic code; any other materials used in the review.	Supplementary Tables 1-3; OSF registration/project materials

Appendix 2 

**Table 3:** Detailed search strategies and search results by database and search method. Detailed search strategies used to identify studies evaluating sodium-glucose cotransporter 2 (SGLT2) inhibitors and glucagon-like peptide-1 receptor agonists (GLP-1 RAs) in type 2 diabetes mellitus. Searches included class-specific strategies and strategies targeting direct comparisons or combination therapy across PubMed/MEDLINE, Embase, and Cochrane CENTRAL, with Google Scholar and backward and forward citation searching used as additional search methods. The table reports the exact search syntax, the number of results retrieved, and the number of records screened or exported for each search. Google Scholar screening was limited to the first 200 results from each search. ASCVD: Atherosclerotic cardiovascular disease; CKD: Chronic kidney disease; eGFR: Estimated glomerular filtration rate; GLP-1 RAs: Glucagon-like peptide-1 receptor agonists; HbA1c: Glycated hemoglobin; MACE: Major adverse cardiovascular events; SGLT2: Sodium-glucose cotransporter 2; T2DM: Type 2 diabetes mellitus.

Search	Source / purpose	Exact search strategy	Results	Screened / exported
S01	PubMed/MEDLINE (PubMed) SGLT2 class-specific	("type 2 diabetes"[Title/Abstract] OR "type 2 diabetes mellitus"[Title/Abstract] OR T2DM[Title/Abstract]) AND (SGLT2[Title/Abstract] OR SGLT-2[Title/Abstract] OR "SGLT2 inhibitor"[Title/Abstract] OR "SGLT2 inhibitors"[Title/Abstract] OR "sodium-glucose cotransporter 2 inhibitor"[Title/Abstract] OR "sodium-glucose cotransporter 2 inhibitors"[Title/Abstract] OR empagliflozin[Title/Abstract] OR canagliflozin[Title/Abstract] OR dapagliflozin[Title/Abstract] OR ertugliflozin[Title/Abstract]) AND (cardiovascular[Title/Abstract] OR "cardiovascular disease"[Title/Abstract] OR MACE[Title/Abstract] OR "major adverse cardiovascular events"[Title/Abstract] OR ASCVD[Title/Abstract] OR "atherosclerotic cardiovascular disease"[Title/Abstract] OR "heart failure"[Title/Abstract] OR "heart failure hospitalization"[Title/Abstract] OR stroke[Title/Abstract] OR mortality[Title/Abstract] OR "cardiovascular death"[Title/Abstract] OR renal[Title/Abstract] OR kidney[Title/Abstract] OR CKD[Title/Abstract] OR "chronic kidney disease"[Title/Abstract] OR eGFR[Title/Abstract] OR "kidney failure"[Title/Abstract] OR "kidney disease progression"[Title/Abstract] OR safety[Title/Abstract] OR "adverse events"[Title/Abstract])	7,202	7,202
S02	PubMed/MEDLINE (PubMed) GLP-1 RA class-specific	("type 2 diabetes"[Title/Abstract] OR "type 2 diabetes mellitus"[Title/Abstract] OR T2DM[Title/Abstract]) AND ("GLP-1"[Title/Abstract] OR GLP1[Title/Abstract] OR "GLP-1 receptor agonist"[Title/Abstract] OR "GLP-1 receptor agonists"[Title/Abstract] OR "glucagon-like peptide-1 receptor agonist"[Title/Abstract] OR "glucagon-like peptide-1 receptor agonists"[Title/Abstract] OR liraglutide[Title/Abstract] OR semaglutide[Title/Abstract] OR dulaglutide[Title/Abstract] OR exenatide[Title/Abstract] OR lixisenatide[Title/Abstract] OR albiglutide[Title/Abstract]) AND (cardiovascular[Title/Abstract] OR "cardiovascular disease"[Title/Abstract] OR MACE[Title/Abstract] OR "major adverse cardiovascular events"[Title/Abstract] OR ASCVD[Title/Abstract] OR "atherosclerotic cardiovascular disease"[Title/Abstract] OR "heart failure"[Title/Abstract] OR "heart failure hospitalization"[Title/Abstract] OR stroke[Title/Abstract] OR mortality[Title/Abstract] OR "cardiovascular death"[Title/Abstract] OR renal[Title/Abstract] OR kidney[Title/Abstract] OR CKD[Title/Abstract] OR "chronic kidney disease"[Title/Abstract] OR eGFR[Title/Abstract] OR "kidney failure"[Title/Abstract] OR "kidney disease progression"[Title/Abstract] OR safety[Title/Abstract] OR "adverse events"[Title/Abstract])	7,097	7,097
S03	PubMed/MEDLINE (PubMed) Direct comparison/combination	("type 2 diabetes"[Title/Abstract] OR "type 2 diabetes mellitus"[Title/Abstract] OR T2DM[Title/Abstract]) AND (SGLT2[Title/Abstract] OR SGLT-2[Title/Abstract] OR "SGLT2 inhibitor"[Title/Abstract] OR "SGLT2 inhibitors"[Title/Abstract] OR empagliflozin[Title/Abstract] OR canagliflozin[Title/Abstract] OR dapagliflozin[Title/Abstract] OR ertugliflozin[Title/Abstract]) AND ("GLP-1"[Title/Abstract] OR GLP1[Title/Abstract] OR "GLP-1 receptor agonist"[Title/Abstract] OR "GLP-1 receptor agonists"[Title/Abstract] OR liraglutide[Title/Abstract] OR semaglutide[Title/Abstract] OR dulaglutide[Title/Abstract] OR exenatide[Title/Abstract] OR lixisenatide[Title/Abstract] OR albiglutide[Title/Abstract]) AND (cardiovascular[Title/Abstract] OR MACE[Title/Abstract] OR "heart failure"[Title/Abstract] OR stroke[Title/Abstract] OR mortality[Title/Abstract] OR renal[Title/Abstract] OR kidney[Title/Abstract] OR CKD[Title/Abstract] OR eGFR[Title/Abstract] OR HbA1c[Title/Abstract] OR weight[Title/Abstract] OR safety[Title/Abstract] OR "adverse events"[Title/Abstract])	1,475	1,475
S04	Embase (Embase.com / institutional) SGLT2 class-specific	("type 2 diabetes":ti,ab OR "type 2 diabetes mellitus":ti,ab OR T2DM:ti,ab) AND (SGLT2:ti,ab OR SGLT-2:ti,ab OR "SGLT2 inhibitor":ti,ab OR "SGLT2 inhibitors":ti,ab OR "sodium-glucose cotransporter 2 inhibitor":ti,ab OR "sodium-glucose cotransporter 2 inhibitors":ti,ab OR empagliflozin:ti,ab OR canagliflozin:ti,ab OR dapagliflozin:ti,ab OR ertugliflozin:ti,ab) AND (cardiovascular:ti,ab OR "cardiovascular disease":ti,ab OR MACE:ti,ab OR "major adverse cardiovascular events":ti,ab OR ASCVD:ti,ab OR "atherosclerotic cardiovascular disease":ti,ab OR "heart failure":ti,ab OR "heart failure hospitalization":ti,ab OR stroke:ti,ab OR mortality:ti,ab OR "cardiovascular death":ti,ab OR renal:ti,ab OR kidney:ti,ab OR CKD:ti,ab OR "chronic kidney disease":ti,ab OR eGFR:ti,ab OR "kidney failure":ti,ab OR "kidney disease progression":ti,ab OR safety:ti,ab OR "adverse events":ti,ab)	12,575	12,575
S05	Embase (Embase.com / institutional) GLP-1 RA class-specific	("type 2 diabetes":ti,ab OR "type 2 diabetes mellitus":ti,ab OR T2DM:ti,ab) AND ("GLP-1":ti,ab OR GLP1:ti,ab OR "GLP-1 receptor agonist":ti,ab OR "GLP-1 receptor agonists":ti,ab OR "glucagon-like peptide-1 receptor agonist":ti,ab OR "glucagon-like peptide-1 receptor agonists":ti,ab OR liraglutide:ti,ab OR semaglutide:ti,ab OR dulaglutide:ti,ab OR exenatide:ti,ab OR lixisenatide:ti,ab OR albiglutide:ti,ab) AND (cardiovascular:ti,ab OR "cardiovascular disease":ti,ab OR MACE:ti,ab OR "major adverse cardiovascular events":ti,ab OR ASCVD:ti,ab OR "atherosclerotic cardiovascular disease":ti,ab OR "heart failure":ti,ab OR "heart failure hospitalization":ti,ab OR stroke:ti,ab OR mortality:ti,ab OR "cardiovascular death":ti,ab OR renal:ti,ab OR kidney:ti,ab OR CKD:ti,ab OR "chronic kidney disease":ti,ab OR eGFR:ti,ab OR "kidney failure":ti,ab OR "kidney disease progression":ti,ab OR safety:ti,ab OR "adverse events":ti,ab)	12,846	12,846
S06	Embase (Embase.com / institutional) Direct comparison/combination	("type 2 diabetes":ti,ab OR "type 2 diabetes mellitus":ti,ab OR T2DM:ti,ab) AND (SGLT2:ti,ab OR SGLT-2:ti,ab OR "SGLT2 inhibitor":ti,ab OR "SGLT2 inhibitors":ti,ab OR empagliflozin:ti,ab OR canagliflozin:ti,ab OR dapagliflozin:ti,ab OR ertugliflozin:ti,ab) AND ("GLP-1":ti,ab OR GLP1:ti,ab OR "GLP-1 receptor agonist":ti,ab OR "GLP-1 receptor agonists":ti,ab OR liraglutide:ti,ab OR semaglutide:ti,ab OR dulaglutide:ti,ab OR exenatide:ti,ab OR lixisenatide:ti,ab OR albiglutide:ti,ab) AND (cardiovascular:ti,ab OR MACE:ti,ab OR "heart failure":ti,ab OR stroke:ti,ab OR mortality:ti,ab OR renal:ti,ab OR kidney:ti,ab OR CKD:ti,ab OR eGFR:ti,ab OR HbA1c:ti,ab OR weight:ti,ab OR safety:ti,ab OR "adverse events":ti,ab)	2,487	2,487
S07	Cochrane CENTRAL (Cochrane Library) SGLT2 class-specific	("type 2 diabetes":ti,ab OR "type 2 diabetes mellitus":ti,ab OR T2DM:ti,ab) AND (SGLT2:ti,ab OR SGLT-2:ti,ab OR "SGLT2 inhibitor":ti,ab OR "SGLT2 inhibitors":ti,ab OR "sodium-glucose cotransporter 2 inhibitor":ti,ab OR "sodium-glucose cotransporter 2 inhibitors":ti,ab OR empagliflozin:ti,ab OR canagliflozin:ti,ab OR dapagliflozin:ti,ab OR ertugliflozin:ti,ab) AND (cardiovascular:ti,ab OR "cardiovascular disease":ti,ab OR MACE:ti,ab OR "major adverse cardiovascular events":ti,ab OR ASCVD:ti,ab OR "atherosclerotic cardiovascular disease":ti,ab OR "heart failure":ti,ab OR "heart failure hospitalization":ti,ab OR stroke:ti,ab OR mortality:ti,ab OR "cardiovascular death":ti,ab OR renal:ti,ab OR kidney:ti,ab OR CKD:ti,ab OR "chronic kidney disease":ti,ab OR eGFR:ti,ab OR "kidney failure":ti,ab OR "kidney disease progression":ti,ab OR safety:ti,ab OR "adverse events":ti,ab)	1,250	1,250
S08	Cochrane CENTRAL (Cochrane Library) GLP-1 RA class-specific	("type 2 diabetes":ti,ab OR "type 2 diabetes mellitus":ti,ab OR T2DM:ti,ab) AND ("GLP-1":ti,ab OR GLP1:ti,ab OR "GLP-1 receptor agonist":ti,ab OR "GLP-1 receptor agonists":ti,ab OR "glucagon-like peptide-1 receptor agonist":ti,ab OR "glucagon-like peptide-1 receptor agonists":ti,ab OR liraglutide:ti,ab OR semaglutide:ti,ab OR dulaglutide:ti,ab OR exenatide:ti,ab OR lixisenatide:ti,ab OR albiglutide:ti,ab) AND (cardiovascular:ti,ab OR "cardiovascular disease":ti,ab OR MACE:ti,ab OR "major adverse cardiovascular events":ti,ab OR ASCVD:ti,ab OR "atherosclerotic cardiovascular disease":ti,ab OR "heart failure":ti,ab OR "heart failure hospitalization":ti,ab OR stroke:ti,ab OR mortality:ti,ab OR "cardiovascular death":ti,ab OR renal:ti,ab OR kidney:ti,ab OR CKD:ti,ab OR "chronic kidney disease":ti,ab OR eGFR:ti,ab OR "kidney failure":ti,ab OR "kidney disease progression":ti,ab OR safety:ti,ab OR "adverse events":ti,ab)	1,278	1,278
S09	Cochrane CENTRAL (Cochrane Library) Direct comparison/combination	("type 2 diabetes":ti,ab OR "type 2 diabetes mellitus":ti,ab OR T2DM:ti,ab) AND (SGLT2:ti,ab OR SGLT-2:ti,ab OR "SGLT2 inhibitor":ti,ab OR "SGLT2 inhibitors":ti,ab OR empagliflozin:ti,ab OR canagliflozin:ti,ab OR dapagliflozin:ti,ab OR ertugliflozin:ti,ab) AND ("GLP-1":ti,ab OR GLP1:ti,ab OR "GLP-1 receptor agonist":ti,ab OR "GLP-1 receptor agonists":ti,ab OR liraglutide:ti,ab OR semaglutide:ti,ab OR dulaglutide:ti,ab OR exenatide:ti,ab OR lixisenatide:ti,ab OR albiglutide:ti,ab) AND (cardiovascular:ti,ab OR MACE:ti,ab OR "heart failure":ti,ab OR stroke:ti,ab OR mortality:ti,ab OR renal:ti,ab OR kidney:ti,ab OR CKD:ti,ab OR eGFR:ti,ab OR HbA1c:ti,ab OR weight:ti,ab OR safety:ti,ab OR "adverse events":ti,ab)	59	59
S10	Google Scholar (Google Scholar) SGLT2 class-specific	"type 2 diabetes" SGLT2 cardiovascular renal kidney "heart failure" MACE mortality	6,590	200
S11	Google Scholar (Google Scholar) GLP-1 RA class-specific	"type 2 diabetes" "GLP-1 receptor agonist" cardiovascular renal kidney MACE stroke mortality	2,180	200
S12	Google Scholar (Google Scholar) Direct comparison/combination	"type 2 diabetes" SGLT2 "GLP-1 receptor agonist" cardiovascular renal kidney combination	5,780	200
S13	Other (Reference lists) Backward citation searching	Backward citation searching of reference lists of relevant studies.	47	47
S14	Other (Citation index) Forward citation searching	Forward citation searching using a citation index. These records were included within the aggregate backward/forward citation-search count.	Included in aggregate 47	Included in aggregate 47

Appendix 3 

**Table 4:** Study-level risk-of-bias and methodological quality assessments. Study-level risk-of-bias and methodological quality assessments for studies included in the systematic review. Randomized controlled trials and secondary analyses of randomized trials were assessed using the Cochrane Risk of Bias 2 (RoB 2) tool; nonrandomized comparative studies were assessed using the Risk Of Bias In Non-randomized Studies of Interventions (ROBINS-I) tool; and systematic reviews and meta-analyses were assessed using A Measurement Tool to Assess Systematic Reviews 2 (AMSTAR 2). Panels A-C present assessments according to study design and the corresponding appraisal tool. Background and contextual references not included in the systematic evidence synthesis were not assessed. AMSTAR 2: A Measurement Tool to Assess Systematic Reviews 2; RCT: Randomized controlled trial; RoB 2: Risk of Bias 2; ROBINS-I: Risk Of Bias In Non-randomized Studies of Interventions; SGLT2: sodium-glucose cotransporter 2.

Study	Study design / evidence type	Tool	Overall judgment	Principal basis / concern
Panel A. Randomized controlled trials and secondary analyses				
EMPA-REG OUTCOME (Zinman B, et al. [6])	Randomized controlled trial	RoB 2	Low risk	Dedicated cardiovascular outcome trial
DECLARE-TIMI 58 (Wiviott SD, et al. [8])	Randomized controlled trial	RoB 2	Low risk	Dedicated cardiovascular outcome trial
DAPA-HF (McMurray JJ, et al. [9])	Randomized controlled trial	RoB 2	Low risk	Dedicated heart failure outcome trial
EMPEROR-Reduced (Packer M, et al. [10])	Randomized controlled trial	RoB 2	Low risk	Dedicated heart failure outcome trial
LEADER (Marso SP, et al. [15])	Randomized controlled trial	RoB 2	Low risk	Dedicated cardiovascular outcome trial
SUSTAIN-6 (Marso SP, et al. [16])	Randomized controlled trial	RoB 2	Low risk	Dedicated cardiovascular outcome trial
CREDENCE (Perkovic V, et al. [12])	Randomized controlled trial	RoB 2	Low risk	Dedicated kidney outcome trial
DAPA-CKD (Heerspink HJ, et al. [13])	Randomized controlled trial	RoB 2	Low risk	Dedicated kidney outcome trial
EMPA-KIDNEY (Herrington WG, et al. [14])	Randomized controlled trial	RoB 2	Low risk	Dedicated kidney outcome trial
DELIVER (Solomon SD, et al. [11])	Randomized controlled trial	RoB 2	Low risk	Dedicated heart failure outcome trial
FLOW (Perkovic V, et al. [17])	Randomized controlled trial	RoB 2	Low risk	Dedicated kidney outcome trial
CANVAS (Rajagopalan S and Brook R [7])	Randomized controlled trial	RoB 2	Some concerns	Interim unblinding and integrated-program pooling
SOUL post hoc analysis (Mulvagh SL, et al. [45])	Secondary/post hoc RCT analysis	RoB 2	Some concerns	Secondary analysis not powered for the reanalyzed endpoint
FLOW subgroup analysis (Mann JF, et al. [26])	Secondary/subgroup RCT analysis	RoB 2	Some concerns	Subgroup analysis of concomitant SGLT2 inhibitor use
Panel B. Nonrandomized comparative studies				
Blum MF, et al. [27]	Target-trial emulation	ROBINS-I	Moderate risk	Residual confounding despite target-trial emulation
Hung A, et al. [39]	Target-trial emulation	ROBINS-I	Moderate risk	Residual confounding despite target-trial emulation
Patorno E, et al. [34]	Propensity-matched cohort	ROBINS-I	Moderate risk	Residual confounding in comparative cohort
Ueda P, et al. [32]	Comparative cohort	ROBINS-I	Moderate risk	Residual confounding in comparative cohort
Lugner M, et al. [35]	Nationwide cohort	ROBINS-I	Moderate risk	Residual confounding in nationwide observational comparison
Edmonston D, et al. [33]	Comparative effectiveness cohort	ROBINS-I	Moderate risk	Residual confounding despite adjusted comparative design
Simms-Williams N, et al. [28]	Population-based cohort	ROBINS-I	Moderate risk	Residual confounding in population-based cohort
Bu F, et al. [37]	Comparative effectiveness cohort	ROBINS-I	Moderate risk	Residual confounding in comparative effectiveness analysis
Sun W, et al. [43]	Propensity-matched cohort	ROBINS-I	Serious risk	Greater potential for confounding by indication, prevalent-user design, or limited adjustment
Chuang MH, et al. [44]	Comparative cohort	ROBINS-I	Serious risk	Greater potential for confounding by indication or limited adjustment
Tang H, et al. [47]	Comparative cohort	ROBINS-I	Serious risk	Greater potential for confounding by indication or limited adjustment
Hartsell SE, et al. [36]	Comparative cohort	ROBINS-I	Serious risk	Greater potential for residual confounding or limited adjustment
Panel C. Systematic reviews and meta-analyses				
Apperloo EM, et al. [22]	Systematic review/meta-analysis	AMSTAR 2	High confidence	Rigorous collaborative, protocol-registered review
Neuen BL, et al. [40]	Systematic review/meta-analysis	AMSTAR 2	High confidence	Rigorous, protocol-registered review
Palmer SC, et al. [29]	Systematic review/network meta-analysis	AMSTAR 2	High confidence	Rigorous, protocol-registered network meta-analysis
Badve SV, et al. [38]	Systematic review/meta-analysis	AMSTAR 2	High confidence	Rigorous, protocol-registered review
Zelniker TA, et al. [31]	Systematic review/meta-analysis	AMSTAR 2	Moderate confidence	Comprehensive comparative meta-analysis; noncritical reporting limitations
Hussein H, et al. [30]	Systematic review/network meta-analysis	AMSTAR 2	Moderate confidence	Well-conducted network meta-analysis with noncritical reporting limitations
Kunutsor SK, et al. [41]	Systematic review/meta-analysis	AMSTAR 2	Moderate confidence	Registered cardiovascular outcome trial meta-analysis; noncritical weaknesses
Colombijn JM, et al. [24]	Systematic review/meta-analysis of cohort studies	AMSTAR 2	Moderate confidence	Protocol-based review with noncritical reporting gaps
Castellana M, et al. [23]	Systematic review/meta-analysis	AMSTAR 2	Low confidence	Critical weakness related to absence of a registered a priori protocol
Li C, et al. [25]	Systematic review/meta-analysis	AMSTAR 2	Low confidence	Critical weakness related to protocol/excluded-study documentation
Schousboe JT, et al. [48]	Systematic review/meta-analysis	AMSTAR 2	Critically low confidence	Multiple critical domains unmet for an intervention-effect appraisal
Wang Y, et al. [42]	Systematic review/meta-analysis	AMSTAR 2	Critically low confidence	Multiple critical weaknesses, including no a priori protocol and no excluded-study list

Appendix 4 

**Table 5:** GRADE summary of findings for principal comparative outcomes. Summary of findings using the Grading of Recommendations Assessment, Development and Evaluation (GRADE) framework for principal comparative cardiovascular, kidney, mortality, safety, and combination-therapy outcomes. Certainty was assessed at the outcome level using the GRADE domains of risk of bias, inconsistency, indirectness, imprecision, and publication bias. Randomized and nonrandomized evidence streams were considered according to their contribution to each outcome. Because this systematic review did not perform a de novo meta-analysis, no new pooled relative or absolute effect estimates were calculated; the table summarizes the direction of effect and the certainty of the body of evidence. GRADE: Grading of Recommendations Assessment, Development and Evaluation; GLP-1: Glucagon-like peptide-1; MACE: Major adverse cardiovascular events; NRSI: Nonrandomized studies of interventions; RCT: Randomized controlled trial; ROBINS-I: Risk of Bias In Non-randomized Studies of Interventions; SGLT2: Sodium-glucose cotransporter 2.

Outcome	Predominant evidence stream	Direction / interpretation	GRADE certainty	Main downgrading domain(s)	Rationale
MACE (composite)	Placebo-anchored RCT meta-analyses supplemented by comparative cohorts	Both classes reduce composite MACE versus placebo; no significant between-class difference in composite MACE, with differing cardiovascular profiles (GLP-1 receptor agonists favored for atherosclerotic events and SGLT2 inhibitors for heart failure)	Moderate	Indirectness (downgraded one level)	Few direct head-to-head randomized comparisons; most comparative inference is indirect. Direct network estimates show no significant difference in three-point MACE between classes (Hussein et al., Diabetes Obes Metab, 2020).
Heart failure hospitalization	RCT meta-analyses and dedicated heart failure trials	SGLT2 inhibitors favored, with consistent reduction in heart failure hospitalization	Moderate	Indirectness (downgraded one level)	Strong and consistent randomized evidence, but most between-class comparisons are indirect.
Nonfatal stroke	RCT meta-analyses	GLP-1 receptor agonists favored	Moderate	Imprecision (downgraded one level)	Direction is relatively consistent, but lower event frequency and comparative precision limit certainty.
Kidney disease progression / kidney failure	Dedicated kidney RCTs, meta-analyses, and target-trial emulations	SGLT2 inhibitors generally favored for kidney disease progression; GLP-1 receptor agonists also provide kidney benefit	Moderate	Indirectness (downgraded one level)	Dedicated trials support both classes, but direct randomized class-to-class comparisons remain limited. Within-class certainty is high that each class reduces kidney disease progression versus placebo (CREDENCE, DAPA-CKD, and EMPA-KIDNEY for SGLT2 inhibitors; FLOW for semaglutide); the moderate rating applies to the between-class comparison, which rests on indirect and target-trial-emulation evidence rather than direct randomization. The larger SGLT2 inhibitor effect on progression reflects a class-related difference rather than unexplained inconsistency, so inconsistency was not additionally invoked (Palmer et al., BMJ, 2021; Neuen et al., Circulation, 2024).
All-cause and cardiovascular mortality	RCTs and RCT meta-analyses	Benefits demonstrated in selected high-risk populations; no consistent between-class superiority	Moderate	Indirectness (downgraded one level)	Mortality estimates arise largely from separate trial programs rather than direct randomized comparisons.
Comparative effectiveness in real-world subgroups	ROBINS-I comparative cohorts and target-trial emulations	Outcome-specific differences observed between classes	Low	Risk of bias (downgraded two levels)	NRSI stream entered GRADE at high certainty (ROBINS-I appraised) and was downgraded two levels for residual confounding; four nonrandomized comparative studies were judged to be at serious risk of bias.
Genital infections	Randomized trial meta-analytic evidence	Higher risk with SGLT2 inhibitors	High	None	Large, consistent, precise class-specific signal with a direct between-class comparator difference; indirectness does not materially threaten the estimate (Palmer et al., BMJ, 2021).
Severe gastrointestinal adverse effects	Randomized trial meta-analytic evidence	Higher risk with GLP-1 receptor agonists	Low	Risk of bias and imprecision (downgraded two levels, one per domain)	Downgraded one level for risk of bias (heterogeneous event definitions and ascertainment across trials) and one level for imprecision (low event frequency and wide comparative estimates), for a two-level reduction from a high starting point.
Combination therapy: cardiovascular and kidney outcomes	RCT subgroup/meta-analytic evidence supplemented by observational cohorts	Potential complementary benefit; superiority over appropriately selected monotherapy not established	Low	Indirectness, risk of bias, and suspected publication bias	Started at high certainty (randomized subgroup/meta-analytic evidence) and downgraded two levels overall: one for the combined limitation of indirectness and risk of bias arising from observational evidence, and one for suspected publication bias given the small number of direct combination studies (no formal funnel or small-study test was performed because no de novo meta-analysis was undertaken). It was not downgraded to very low because the direction of effect was consistent across the available direct combination evidence.
